# Glucagon-like peptide 1 receptor (GLP-1R) expression by nerve fibres in inflammatory bowel disease and functional effects in cultured neurons

**DOI:** 10.1371/journal.pone.0198024

**Published:** 2018-05-29

**Authors:** Uma Anand, Yiangos Yiangou, Ayesha Akbar, Tom Quick, Anthony MacQuillan, Mike Fox, Marco Sinisi, Yuri E. Korchev, Ben Jones, Steve R. Bloom, Praveen Anand

**Affiliations:** 1 Peripheral Neuropathy Unit, Centre for Clinical Translation, Department of Medicine, Imperial College London, Hammersmith Hospital, London, United Kingdom; 2 Nanomedicine Research Laboratory, Department of Medicine, Imperial College London, Hammersmith Hospital, London, United Kingdom; 3 Peripheral Nerve Injury Unit, Royal National Orthopaedic Hospital, Stanmore, Middlesex, United Kingdom; 4 Division of Diabetes, Endocrinology and Metabolism, Department of Medicine, Imperial College London, Hammersmith Hospital, London; Max Delbruck Centrum fur Molekulare Medizin Berlin Buch, GERMANY

## Abstract

**Introduction:**

Glucagon like-peptide 1 receptor (GLP-1R) agonists diminish appetite and may contribute to the weight loss in inflammatory bowel disease (IBD).

**Objectives:**

The aim of this study was to determine, for the first time, the expression of GLP-1R by colon nerve fibres in patients with IBD, and functional effects of its agonists in cultured rat and human sensory neurons.

**Methods:**

GLP-1R and other nerve markers were studied by immunohistochemistry in colon biopsies from patients with IBD (n = 16) and controls (n = 8), human dorsal root ganglia (DRG) tissue, and in GLP-1R transfected HEK293 cells. The morphological effects of incretin hormones oxyntomodulin, exendin-4 and glucagon were studied on neurite extension in cultured DRG neurons, and their functional effects on capsaicin and ATP signalling, using calcium imaging.

**Results:**

Significantly increased numbers of colonic mucosal nerve fibres were observed in IBD biopsies expressing GLP-1R (p = 0.0013), the pan-neuronal marker PGP9.5 (p = 0.0008), and sensory neuropeptide CGRP (p = 0.0014). An increase of GLP-1R positive nerve fibres in IBD colon was confirmed with a different antibody to GLP-1R (p = 0.016). GLP-1R immunostaining was intensely positive in small and medium-sized neurons in human DRG, and in human and rat DRG cultured neurons. Co-localization of GLP-1R expression with neuronal markers in colon and DRG confirmed the neural expression of GLP-1R, and antibody specificity was confirmed in HEK293 cells transfected with the GLP-1R. Treatment with oxyntomodulin, exendin-4 and GLP-1 increased neurite length in cultured neurons compared with controls, but did not stimulate calcium influx directly, or affect capsaicin responses. However, exendin-4 significantly enhanced ATP responses in human DRG neurons.

**Conclusion:**

Our results show that increased GLP-1R innervation in IBD bowel could mediate enhanced visceral afferent signalling, and provide a peripheral target for therapeutic intervention. The differential effect of GLP-1R agonists on capsaicin and ATP responses in neurons suggest they may not affect pain mechanisms mediated by the capsaicin receptor TRPV1, but may enhance the effects of purinergic agonists.

## Introduction

Inflammatory bowel disease (IBD) is an idiopathic chronic, inflammatory disorder of the intestinal tract, afflicting 1 in 400 individuals in the UK and compromising their quality of life. Crohn’s disease (CD) may affect any part of the digestive tract, across the full thickness of the bowel wall, while in ulcerative colitis (UC) the mucosal and submucosal layers of the gut are affected. Features such as weight loss, and chronic abdominal symptoms including pain are common in patients with IBD and irritable bowel syndrome (IBS), corresponding with increased nociceptor innervation and expression of TRPV1 (the heat pain and capsaicin receptor), and other sensory receptors [1–4]. In a recent study, significant weight loss prior to diagnosis (>5% BMI loss) was observed in 57% of subjects with Crohn’s Disease and 51% of subjects with Ulcerative colitis [5]. It is thus likely that factors in the immediate environment of sensory nerves innervating the gut may be responsible for their proliferation and signalling via the gut-brain axis. The potent insulinotropic action of the gut incretin GLP-1 that induces satiety is considered to be at least partly mediated by sensory afferents innervating the gut, as sensory denervation by neonatal capsaicin administration in mice impaired insulinotropic effects of GLP-1 [6, 7]. TNF-α activation of leptin-sensitive vagus nerve mechanoreceptors is proposed to underlie the weight loss observed in a feline model of IBD [8]. These findings suggest that altered sensory signalling may be involved in the pathophysiology of IBD.

The bioactive peptides glucagon, mini-glucagon, oxyntomodulin, and glucagon-like peptides 1 and 2 (GLP-1 and GLP-2) are derived from proglucagon, secreted by the L-cells of the gastrointestinal (GI) tract in response to food intake [9], and by submucosal neural stimulation mediated by voltage-gated Ca^++^ channels [10]. GLP-1 is an incretin hormone that binds its receptor GLP-1R in pancreatic beta cells to stimulate the synthesis and release of insulin [11], and reduce appetite in normal, obese and diabetic subjects [12]. GLP-1R agonists are a newer class of treatment for diabetes [13], inducing a glucose-mediated increase in insulin production by pancreatic β-cells [14, 15], and slowing gastric emptying [16, 17]. Oxyntomodulin (Oxm), a post-translational product of preproglucagon in the intestine and CNS, is an agonist at the GLP-1R, inhibits secretion of gastric acid and pancreatic enzymes, and food intake in the rat [18] and humans [19].

The GLP-1R is widely expressed in normal human tissues, including smooth muscle cells of arteries and arterioles of kidney and lung, β-cells of the pancreas, myocytes of the sino-atrial node in the heart, Brunner’s gland in the duodenum and in myenteric plexus neurons in the human [20–22], mouse [23], and non-human primate gut [24]. Its expression in the brain is reported to regulate appetite, memory and learning [25]. Large glucagon and GLP-1 expressing neurons with dense fibres have been described in the avian retina [26–28]. The agonists of the GLP-1R, GLP-1 and exendin-4 induce neurite outgrowth in PC12 cells by a cAMP mediated NGF independent process that is neuroprotective [29]. However, GLP-1 has a very short half-life of 1.5 minutes, being subject to proteolytic degradation by dipeptidyl peptidase 4 (DPP4) [30]. The GLP-1R is also expressed in DRG neurons and sensory innervation of the gut in mice, providing a signalling route to the CNS following activation by distally released GLP-1 via local and spinal sensory nerves [31, 32], and mediates neurite outgrowth after GLP-1 and exendin-4 treatment [32]. The innervation of the gut plays an important role in nutrient sensing, gut motility and pain signalling via the gut-brain axis. The heat pain and capsaicin receptor TRPV1, and the ATP receptor P2X3 are both expressed in sensory nerve fibres innervating the gut, and their expression is known to be increased in biopsies from patients with painful conditions [1–4]. Nerve fibre density and the expression of sensory and pain receptors e.g. TRPV1 are increased in IBD and IBS. However, while Glucagon like-peptide 1 receptor (GLP-1R) agonists diminish appetite and may contribute to weight loss, the expression of GLP-1R in the gut of IBD patients is not known, or its functional relationship with the increased sensory and nociceptor innervation.

We have therefore examined GLP-1R expression in mucosal nerve fibres of normal and diseased bowel tissues. Our finding of increased levels of GLP-1R and sensory/nociceptor innervation in IBD gut tissues, and presence of GLP-1R in human DRG neurons, led to our studies of functional effects; we investigated the effects of oxyntomodulin, exendin-4 and GLP-1 on neurite length, and sensitivity to capsaicin and ATP, in cultured DRG neurons.

## Materials and methods

### Tissues

Colonoscopic, rectosigmoid biopsies were collected from patients with IBD (n = 16; UC = 9, CD = 7), as previously described.^1^ Control biopsies (n = 8) were from patients who were undergoing colonoscopy for other indications (polyp and cancer surveillance) and otherwise had a normal colon. The study was approved by Hammersmith Hospitals NHS Trust Research Ethics Committee and all participants gave fully informed written consent. ^1^ Patients with IBD in remission and without previous surgery were included, for ease of recruitment. Patient median age (years) (IQR) was 56 (40–66), 8 were male, 2 were smokers, none had diabetes. The patients were symptomatic with quiescent disease, meeting all of the following criteria: symptomatic (ileo-) colonic CD or UC; CDAI (Crohn's Disease Activity Index) <150 or UCDAI (Ulcerative Colitis Disease Activity Index) ≤1; C-reactive protein (CRP) <10 mg/l; erythrocyte sedimentation rate (ESR) <20 mm/h; platelets <450×1012/l; white cell count <11×109/l; faecal calprotectin <50 mg/g; and macroscopically normal mucosa at colonoscopy (including the terminal ileum in CD). Median disease duration (years) (IQR) was 11 (10–20). Patients currently complained of abdominal pain and IBS-type symptoms (bloating, varying bowel habit with diarrhoea and/or constipation) as defined by Rome II criteria. Current treatments were: no treatment n = 5, 5-ASA (5-aminosalicylic acid only) n = 3, 5-ASA and Azathioprine n = 2, Azathioprine n = 1, and Methotrexate n = 1. No patients were taking corticosteroids. We are not aware of any evidence that suggests these medications may have a direct effect on visceral nerve growth or function. The control group (n = 8) consisted of asymptomatic patients without IBD or IBS. Their median age (years) (IQR) was 63 (51–68), 4 were male, none were smokers or had diabetes.

All patients had a colonoscopy with standard bowel preparation (4 litres of Kleanprep). Due to the patchy nature of colonic involvement in CD, patients with previous rectal involvement were selected. None of the patients was currently using topical rectal treatment, and all had macroscopically normal colonoscopies. Four mucosal colonic biopsies were collected using standard biopsy forceps at the rectosigmoid junction to ensure uniform sampling.

Human cervical DRG were obtained from 3 patients undergoing reconstructive nerve surgery, requiring their removal as a necessary part of the surgical repair, with patient consent and approval of the local Research Ethics Committee (Royal National Orthopaedic Hospital, Stanmore, UK).

Tissues were snap-frozen and stored at −70°C, or immersed in fixative (4% (w/v) paraformaldehyde in phosphate-buffered saline (PBS: 0.1 M phosphate; 0.9% (w/v) saline; pH 7.3), then washed in PBS containing 15% (w/v) sucrose and 0.05% (w/v) azide for 1 h, before snap freezing in embedding medium (Tissue-Tek OCT compound, Sakura Finetek, Torrance, California, USA). Frozen tissue sections (15 μm) were collected onto poly-l-lysine-coated (Sigma, Poole, Dorset, UK) glass slides. Unfixed tissue sections were post-fixed in 4% (w/v) paraformaldehyde (for Acris GLP-1R antibody), whilst tissue sections from immersion fixed biopsies were allowed to dry on the slide (for Abcam GLP-1R, CGRP and PGP9.5 antibodies only). Endogenous peroxidase was blocked by incubation in 0.3% (w/v) hydrogen peroxide in methanol. After rehydration in PBS, sections were incubated overnight with primary antibodies. Controls included omission of primary antibodies, or their replacement with preimmune serum.

### Cell transfection

HEK293 cells (ECACC), grown in DMEM supplemented with 10% foetal calf serum and 1% penicillin/streptomycin, were seeded onto glass coverslips. After 24 hours, plasmid DNA encoding human GLP-1R under a CMV promoter (SC118878, Origene) was transfected using Lipofectamine 2000 (Thermo Fisher). After 48 hours, confluent cells were fixed in 4% paraformaldehyde for 30 mins at room temperature before processing for immunohistochemistry.

### Immunohistochemistry and analysis

For tissue immunohistochemistry, GLP-1R antibody ab39072 (Abcam, Cambridge, UK) was used at a final titre of 1:1000, CGRP antibody (Merck Millipore AB59200, Watford, UK) was used at a final titre of 1:4000 and rabbit polyclonal anti-human PGP9.5 antibody (RA95101, UltraClone Ltd, UK), was used at 1:40,000, and neuron-specific mouse anti-β-II tubulin/Tuj1 (R&D Systems, Inc, Oxford, UK) at 1:100. A different GLP-1R antibody (AP31362PU-N, Acris Antibodies GmbH, Herford, Germany), was used at a final titre of 1:100, and a cocktail of monoclonal antibodies to the phosphorylated and non-phosphorylated Neurofilaments of size 22 kDa (Clone N52, Sigma-Aldrich, Dorset, UK) and the 57 kDa type III filament, Peripherin (Novocastra Laboratories, Newcastle, UK) were used at final titres of 1:20,000 and 1:500 respectively, as structural neuronal markers for the frozen post-fixed sections.

For immunostaining specificity, antibodies to Abcam GLP-1R were pre-incubated with human GLP-1R peptide antigen (Abcam ab39071), for 3 hours at room temperature, in the range 10 μg to 100pg diluted (1/1500) antibodies prior to immunostaining in DRG or in IBD full thickness colon. Colon sections were also incubated in the Acris GLP-1R antibody at serial 1:100, 1:200 and 1:500 dilutions. Sites of primary antibody attachment were revealed using nickel-enhanced, avidin-biotin peroxidase (ABC; Vector Laboratories, Peterborough, UK). Sections were counter-stained for nuclei in 0.1% w/v aqueous neutral red, dehydrated and mounted in xylene-based mount (DPX; BDH/Merck, Poole, UK) prior to photomicrography.

For immunohistochemical co-localisation studies, serial sections of colon or avulsed DRG were double-labelled by overnight incubation using a mixture of a monoclonal antibody to neuron-specific mouse anti-β-III tubulin / Tuj1 (dilution 1:1,000) together with the polyclonal antibody to GLP-1R (Abcam, dilution 1: 1,000). Immunoreaction for GLP-1R was first revealed using the standard nickel-enhanced ABC peroxidase method as above to give a grey/black product. The neuronal marker was then incubated with biotinylated anti-mouse antibody for 1 hour followed by incubation with ABC alkaline phosphatase (Vector Labs, UK) for a further 1 hour and the immunoreactivity developed with Fast Red (Sigma-Aldrich Company Ltd, UK) to give a red product. Double-labelled preparations were mounted in Ultramount medium (Vector Labs, UK).

For specificity in GLP-1R transfected cell lines, positive or negative cell lines were fixed with 4% paraformaldehyde. Immunostaining was performed using the 2 different GLP-1R antibodies in these wells (Abcam antibody at 1:1000 and 1:2000; Acris antibody 1:100, 1:200 and 1:500) and after processing as described above, coverslips were dried and mounted on to glass slides using DPX.

Immunoreactive nerve fibres and cell markers were quantified by computerised image analysis (Olympus Analysis Five DP Soft, UK), of digital monochrome images as previously described^1^. The grey-shade detection threshold was set at a constant level to allow detection of positive immunostaining, and the area of highlighted immunoreactivity in the mucosa was obtained as a percentage (% area) of the field scanned. Five fields (x40 objective magnification) per tissue section, chosen at random, were scanned, and the mean values of readings obtained by two independent blinded observers were used for final analysis. For GLP-1R analysis, as the fibres showed up as fine linear fibres, the total numbers of fibres were counted per section and results were expressed as the mean number of fibres/mm^2^. Again, five random fields were used by two independent blinded observers. Data were compared using Mann Whitney U test using Winstat for EXCEL software or GraphPad Prism; p values <0.05 were considered as statistically significant.

### Human DRG neuron cultures

Human DRG neuron cultures were prepared as described previously, with enzyme digestion and mechanical dissociation [33, 34]. Neurons were incubated in Ham’s F12 medium containing 10% heat inactivated fetal calf serum and antibiotics (100 μg/ml each penicillin and streptomycin), with added neurotrophic factors (NGF-100 ng/ml, GDNF and NT3-50 ng/ml), in collagen (50 μg/ml) and laminin (20 μg/ml) coated glass bottom plastic petri dishes (MatTek Corp. USA), at 37 ^o^C, for 48 hours before being studied.

### Adult rat DRG neuron cultures

Bilateral DRG were microdissected from 4 adult, female Wistar rats (250 g, Charles River UK, Margate, Kent, UK), after CO_2_ asphyxiation, and collected in Ham’s F12 medium. Following enzyme digestion, dissociated neurons were incubated in collagen (50 μg/ml) and laminin (20 μg/ml) coated glass bottom plastic petri dishes (MatTek Corp. USA), at a density of approximately 1000 neurons per dish in 2 ml modified BSF2 medium [containing 2% heat inactivated fetal calf serum, 0.1 mg/ml transferrin, 60 ng/ml progesterone, 0.16 μg/ml sodium selenite, 3 mg/ml bovine serum albumen (BSA), penicillin/streptomycin 100 μg/ml each, 16 μg/ml putrescine, 10 μg/ml insulin],without neurotrophic factors for neurite outgrowth studies, and with NTFs (100 ng/ml NGF, 50 ng/ml GDNF) for calcium imaging [33]. All procedures were in keeping with the UK Home Office and NC3Rs guidelines.

### Effect on neurite outgrowth

24 hours after plating, duplicate dishes of rat DRG neurons were treated with 100 nM oxyntomodulin, exendin-4 or GLP1 for 48 hours, or no additive (BSF2 medium), before fixing and immunostaining.

### Immunostaining

Cultured rat and human neurons were fixed with 4% PFA for 20 minutes, and permeabilised in methanol (-20°C, for 3 minutes). Primary antibodies used were mouse anti β tubulin (1:200 Sigma, UK) and rabbit anti GLP-1R (1:500 ab 39072 Abcam) for 45 minutes at room temperature. Secondary antibodies used were goat anti mouse IgG (Alexa Fluor 488, Life Technologies), and goat ant rabbit IgG (Alexa Fluor 546, 1:200 each) for 40 minutes at room temperature. All treatments were followed by 3 x 5 minute PBS washes. Glass bottom coverslips were mounted on glass slides in Citifluor (Citifluor, PA, USA), containing the antifade agent DABCO (Sigma, UK), and sealed with nail varnish. Tiff fluorescence images were acquired with an upright Olympus microscope (BX43, Olympus Medical, Essex, UK), using Cellsence software (Olympus, Japan), and widefield epifluoresence optics, after confirming the absence of immunostaining in negative controls, where the primary antibody was omitted. The longest neurite length was measured from individually identifiable neurons in the different groups, and the average expressed as percent of control.

### Calcium imaging

Functional effects of acute oxyntomodulin, exendin-4 or glucagon treatment (100 nM each) on capsaicin or ATP responses were determined in Fura2 AM (Molecular Probes) loaded neurons in HEPES buffered phenol red free HBSS containing 0.1% BSA as previously described [33,34]. Responses to paired capsaicin stimuli, with or without the added compound, were measured as the maximum change in the 340/380 λex nm ratio from baseline, at 37°C in a humidified environment on an inverted Nikon microscope (Diaphot 300). Images were captured every 2 seconds in each of three channels—brightfield, 340 and 380 nm, and recordings of intracellular changes in bound and unbound Ca^2+^ ratio were obtained before, during and after the addition of test compounds. This provided baseline recordings as well as intracellular changes in Ca^2+^ levels in response to added compounds. Cells were uniformly loaded with the dye and no intracellular compartmentalisation of the loaded dye was observed. Images were acquired with a Hamamatsu Orca CCD Camera and analysed with AQM Advance Kinetic imaging software. Individual cells under study were highlighted as regions of interest for calculating the mean ratios of bound to unbound calcium within the area of interest. In each experiment, neurons were exposed to capsaicin for a maximum of two applications only, first to identify capsaicin sensitivity (using 200 nM capsaicin for 15 seconds), followed by washout and rest period of 40 minutes. The second stimulus of 1 μM capsaicin was used to test the effect of the added compounds after the washout period. Only neurons responding with a rapid and sustained increase in 340/380 ratio were selected for study, after the baseline returned to normal. In separate experiments, ATP stimulation preceded exendin-4 application, followed by a second ATP stimulus.

Capsaicin (Sigma, UK), was dissolved in ethanol at 20 mM concentration, aliquoted and stored at -20 ^o^C, and fresh aliquots were made up to 500x final concentration prior to use. ATP was dissolved in sterile distilled water, aliquoted and stored at -20 ^o^C, and freshly thawed aliquots were used for each experiment. Exendin-4, glucagon, and oxyntomodulin (Human/mouse/rat OXM all have the same sequence) were obtained from Bachem (St Helens, UK), dissolved in distilled water, aliquoted and stored at -20 ^o^C, at 3.5 mM concentration, and freshly thawed prior to use.

Student’s unpaired *t*-test was used to compare between groups and *p<0.05 considered to be statistically significant.

## Results

### Colon biopsies

**Fig 1:**
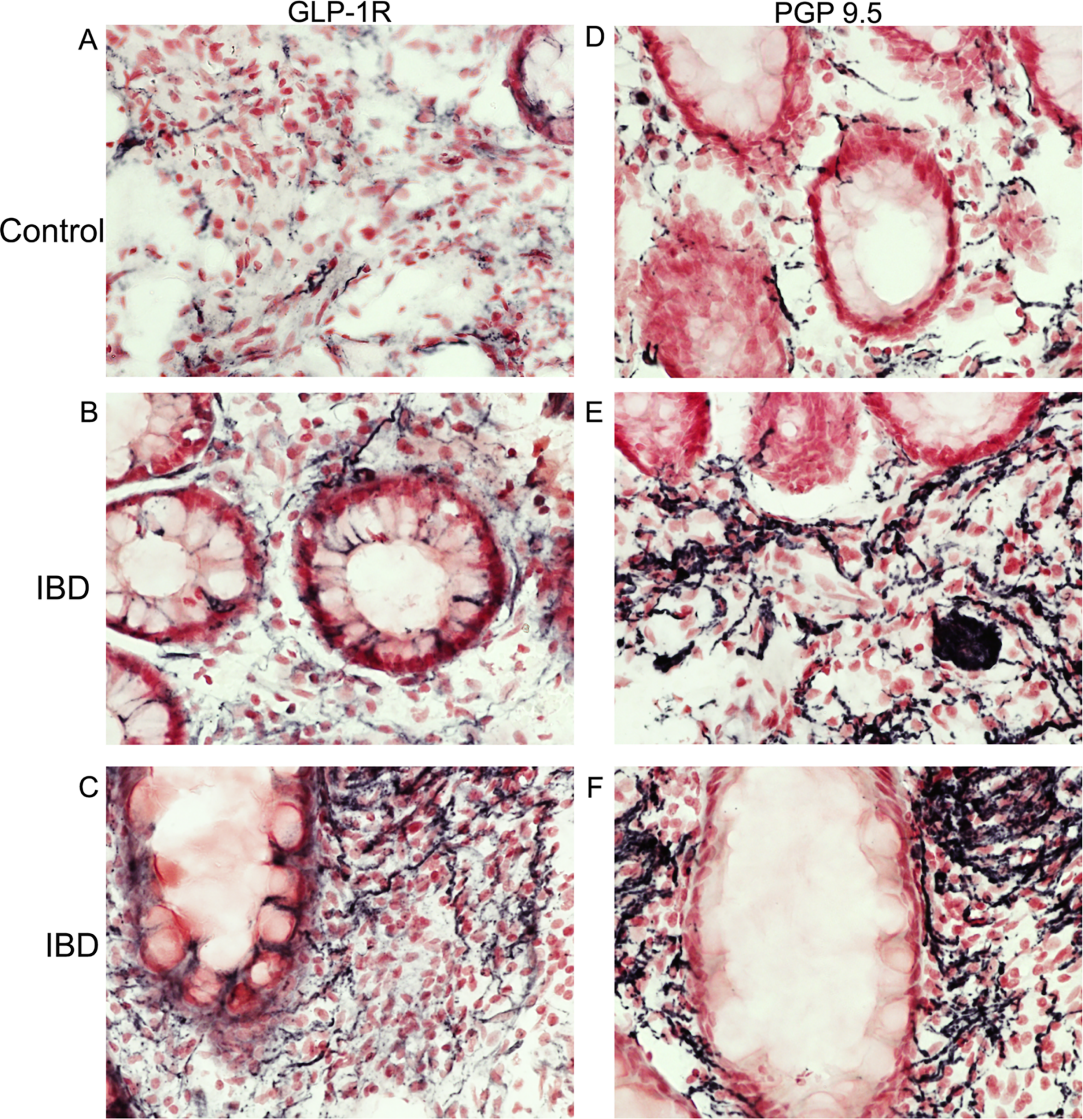
GLP-1Rimmunostaining in colon. GLP-1R positive nerve fibres (Abcam antibody, left panels) and PGP9.5 (right panels) in mucosal sigmoid colon biopsy specimens, from top—control, middle and bottom–IBD, magnification x40. GLP-1R expressing nerve fibres were increased in IBD compared to controls.

In general, few GLP-1R fibres were seen throughout the mucosa of control sigmoid colon biopsy specimens, and appeared greater in the IBD group (Fig 1).

**Fig 2:**
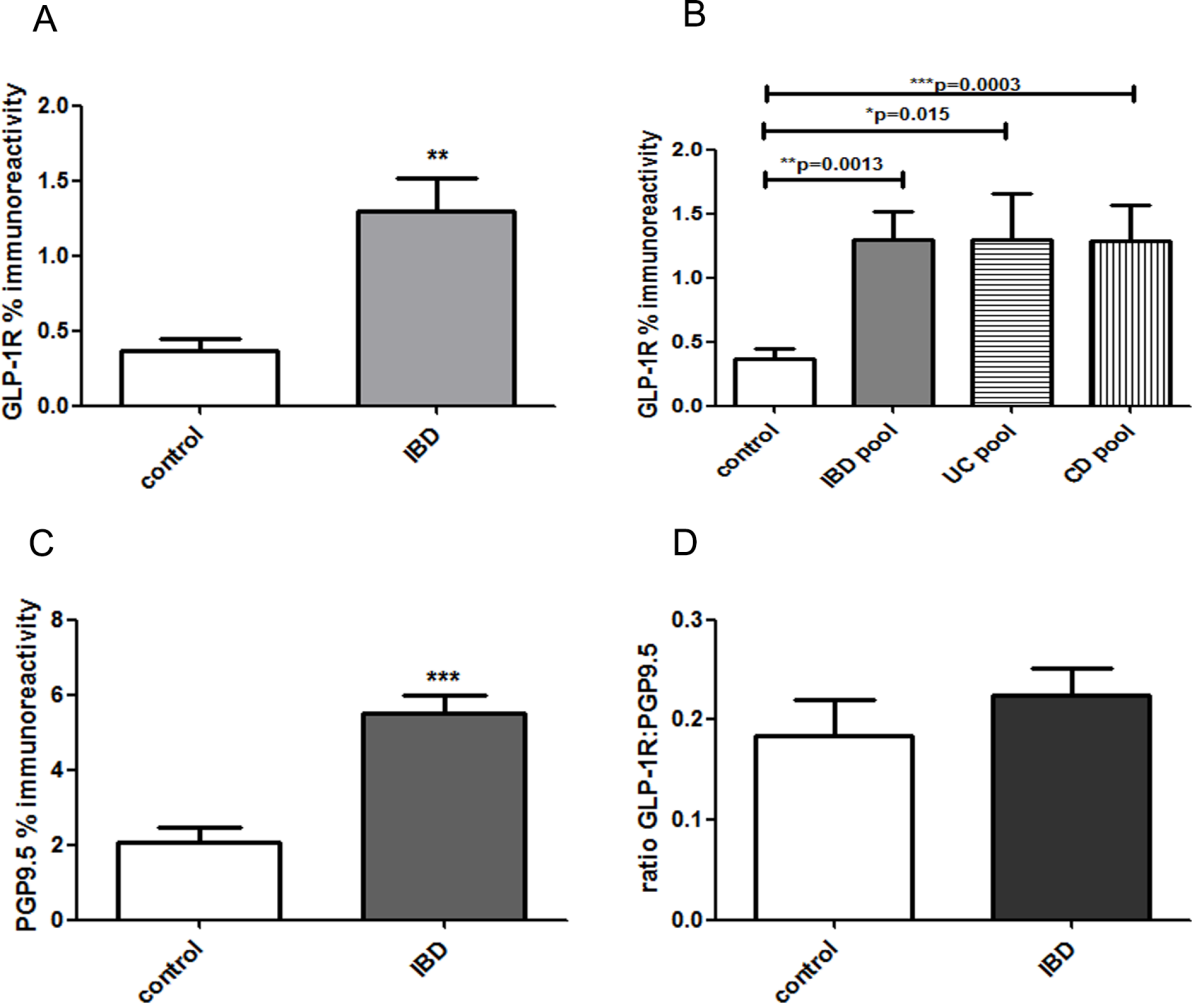
Quantitation of GLP-1R expression in colon. Bar charts showing an overall increase in expression of nerve fibres expressing GLP-1R in the IBD group by image analysis of GLP-1R (A, B) and PGP9.5 (C) in colonic biopsies (mean ± SEM, Mann Whitney U test). There was no difference in the ratio of GLP-1R to PGP9.5, indicating an overall increase in the neuronal fibres expressing GLP-1R.

Image analysis of GLP-1R immunoreactive fibres in sigmoid colon biopsies showed a significant increase in IBD (Fig 2A **p = 0.0013), that were similar in the UC and CD pools (Fig 2B) compared to controls. There was a similar increase with PGP9.5 (Fig 2C ***p = 0.0008) compared to controls; when results were expressed as a ratio there was no statistical difference between the groups (Fig 2D p = 0.6), suggesting an overall increase in expression of neuronal fibres expressing GLP-1R in the IBD group.

**Fig 3:**
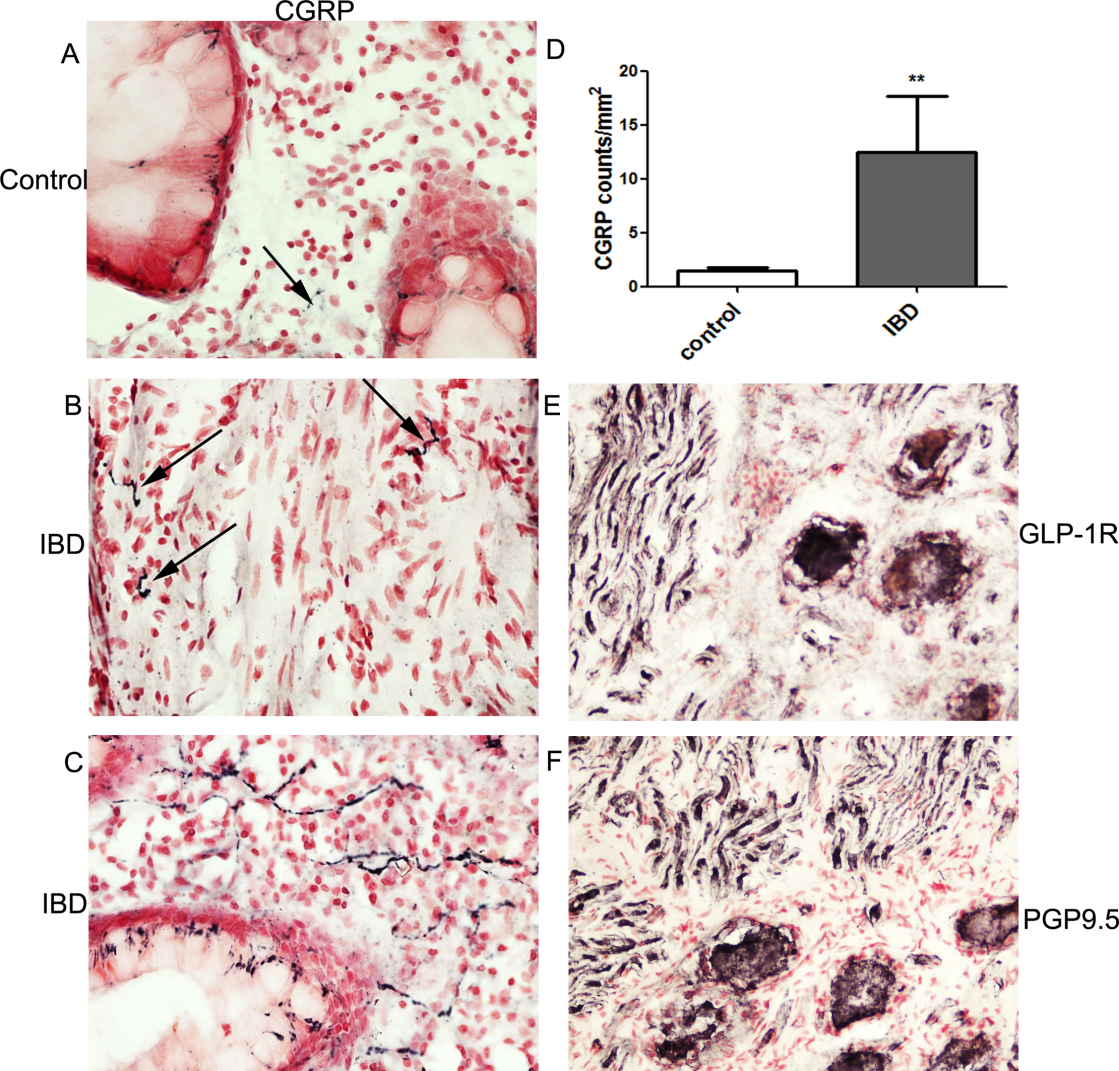
CGRP and GLP-1R immunostaining in colon and DRG. CGRP neuronal staining (arrowed) in mucosal sigmoid colon biopsy specimens from control (A), IBD (B and C), magnification x40. Bar chart showing image analysis of CGRP in colonic biopsies (mean ± SEM, Mann Whitney U test) (D). GLP-1R (Abcam antibody, E), and PGP9.5 (F), staining in avulsion injury DRG, magnification x20.

CGRP fibres were also found in the mucosa of colonic biopsies (Fig 3). Counts of CGRP immunoreactive fibres in sigmoid colon biopsies (expressed as fibres /mm^2^), also showed a significant increase in IBD (**p = 0.0014).

In human DRG tissue, GLP-1R was strongly immunostained in small to medium sized neurons (diameter <50 μm), and nerve fibres/roots (Fig 3E) with the Abcam antibody; immunostaining with the pan-neuronal marker PGP9.5, is also shown in Fig 3F.

**Fig 4:**
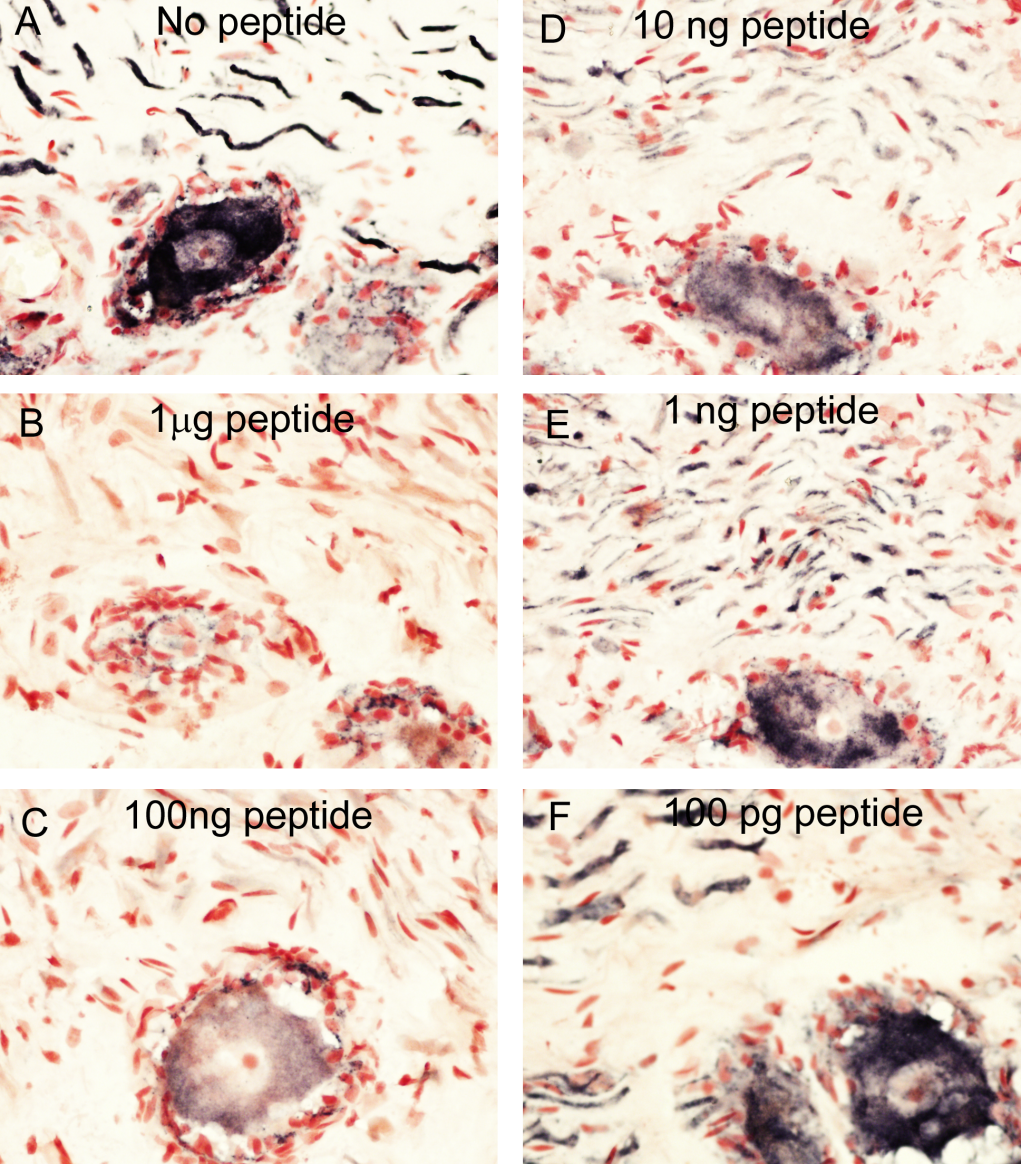
Specificity of GLP-1R immunostaining in DRG tissue. Serial sections of GLP-1R-immunoreactive DRG immunostained with no peptide (A) or with 1 μg peptide (B), 100 ng peptide (C), 10 ng peptide (D), 1 ng peptide (E) or 100 pg peptide (F); Abcam antibody at a dilution of 1:1500; magnification x40.

Pre-absorption of GLP-1R antibody (Abcam) with homologous peptide antigen abolished all neuronal immunostaining in DRG neurons at 10, 1.0 and 0.1 μg peptide antibody at a dilution of 1:1500; gradual return of immunostaining and intensity was observed with decreasing concentrations of peptide antigen (Fig 4).

**Fig 5:**
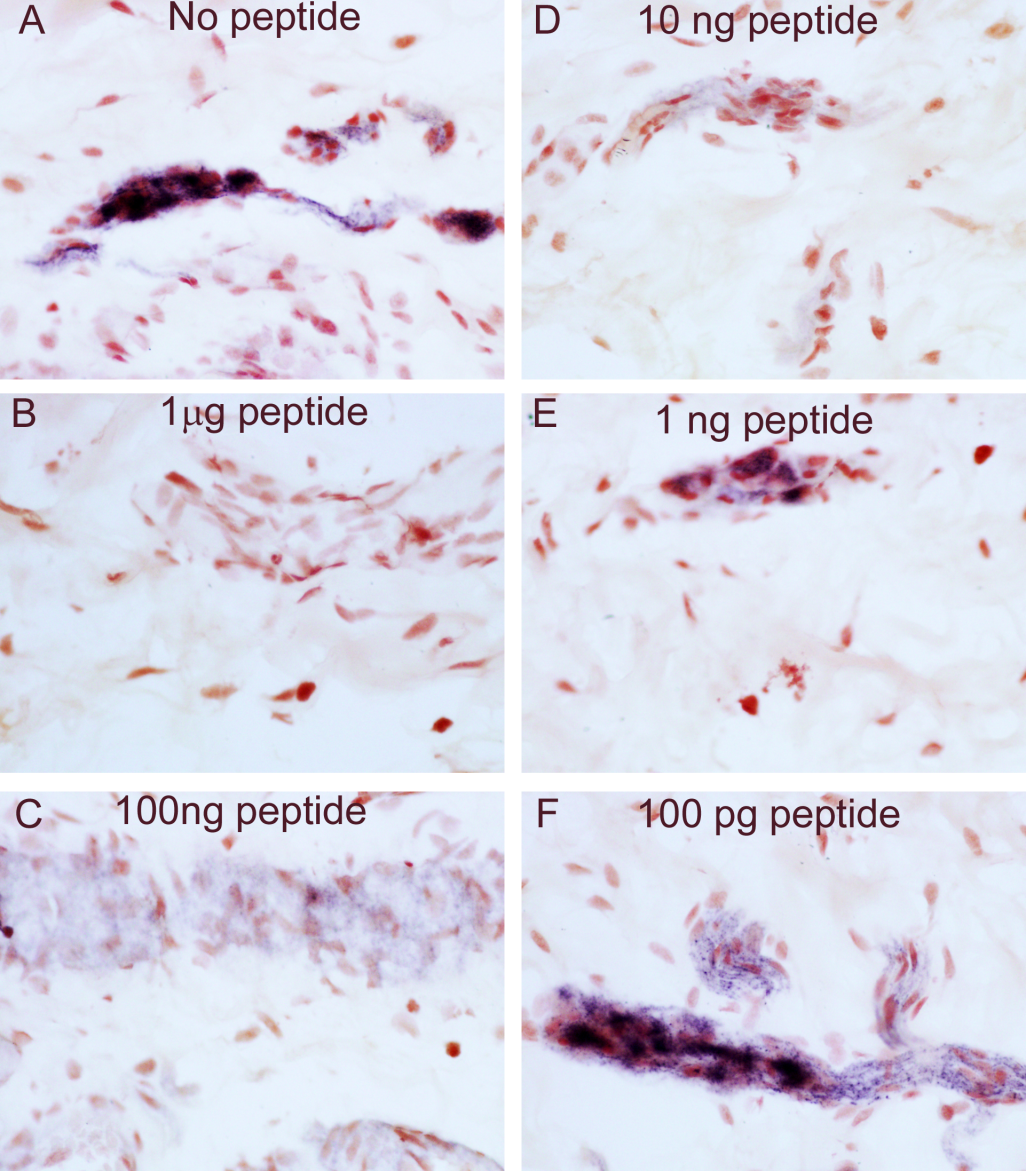
Specificity of GLP-1R immunostaining in colon. Serial sections of GLP-1R immunoreactivity in sub-mucous plexus of a full thickness normal colon immunostained with no peptide (A) or with 1 μg peptide (B), 100 ng peptide (C), 10 ng peptide (D), 1 ng peptide (E) or 100 pg peptide (F) Abcam antibody at a dilution of 1:1500; magnification x40.

Similar results were found when the above studies were performed in IBD colon; preincubation with the peptide abolished all neuronal staining in both the myenteric and sub- mucous plexuses (Fig 5).

No specific staining was seen with either GLP-1R antibody in the HEK negative cell lines, or when primary antibody was omitted. In contrast, in the GLP-1R transfected cell lines both antibodies gave strong staining in these cells, with appropriate serial dilution effects (S1 and S2 Figs).

**Fig 6:**
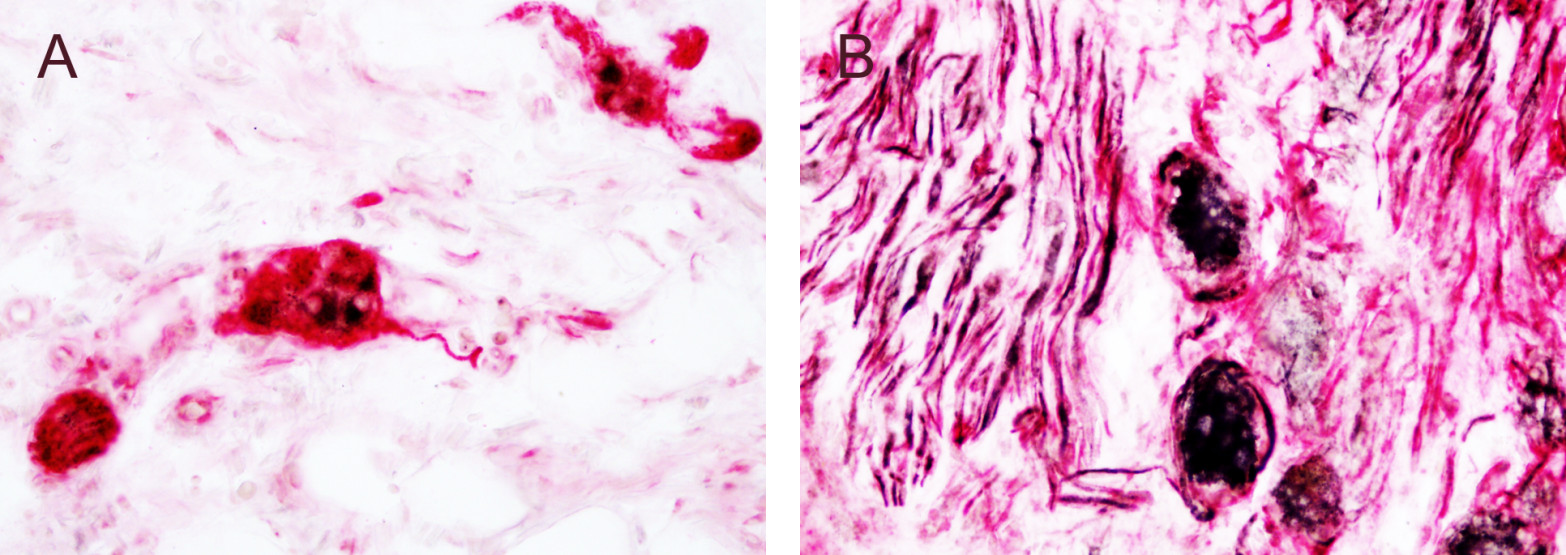
Co-localization of GLP-1R-like immunoreactivity and β tubulin. Double staining of GLP-1R (Abcam GLP-1R antibody at a dilution of 1:1000, black) with the neuronal marker β Tubulin (at a dilution of 1:1000, red) in, A, sub-mucous plexus of colon from a patient with IBD and, B, human DRG; magnification x20.

Double immunostaining with the neuron-specific mouse anti-β-III tubulin / Tuj1 confirmed the neuronal nature of the GLP-1R staining (Fig 6).

**Fig 7:**
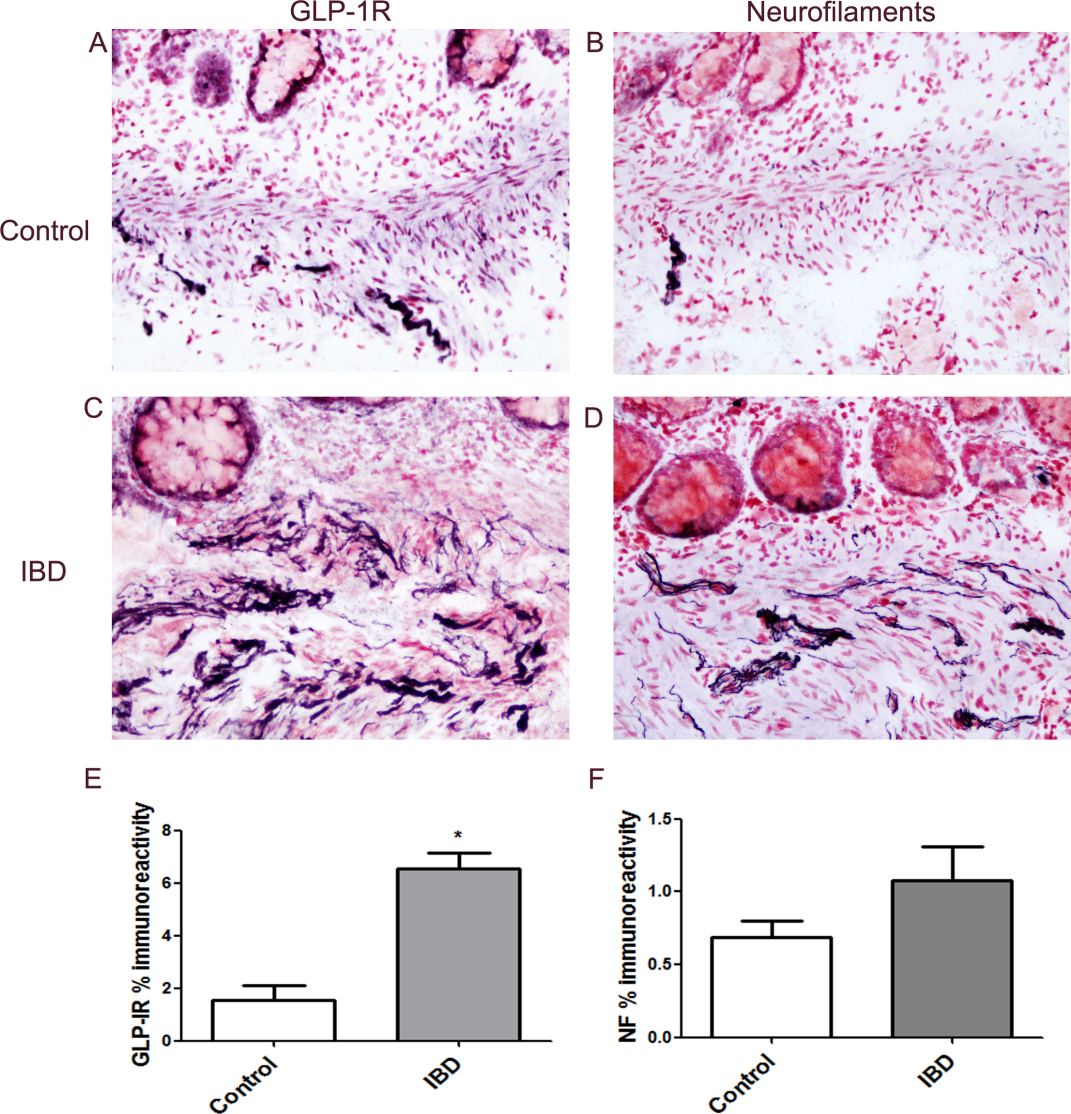
GLP-1R immunostaining in colon. GLP-1R (Acris antibody, A,B) and Neurofilaments (C, D) positive nerve fibres in mucosal sigmoid colon biopsy specimens from top—control and middle–IBD, magnification x20. Bar charts showing an overall increase in expression of neuronal fibres expressing GLP-1R in the IBD group (n = 4) by image analysis of GLP-1R (E) and NF (F) in colonic biopsies (mean ± SEM, Mann Whitney U test).

The different antibody to GLP-1R (Acris) also showed that neuronal staining in colon was greater in IBD compared to controls (Fig 7). Image analysis of sigmoid colon from controls (Mean ± SEM, 0.97 ± 0.40, n = 4) and patients with IBD (4.27 ± 0.91, n = 5) with this antibody confirmed a significant increase in the IBD group (*p = 0.016). Staining with this antibody to GLP-1R (Acris) was best observed at dilution of 1:100; the immunoreactivity diminished with increasing antibody dilution. This antibody detects endogenous levels of total GLP-1R protein, and regarding specificity the synthetic peptide abolished immunofluorescence staining of HeLa cells (Acris source data sheet).

### Cultured DRG neurons

**Fig 8:**
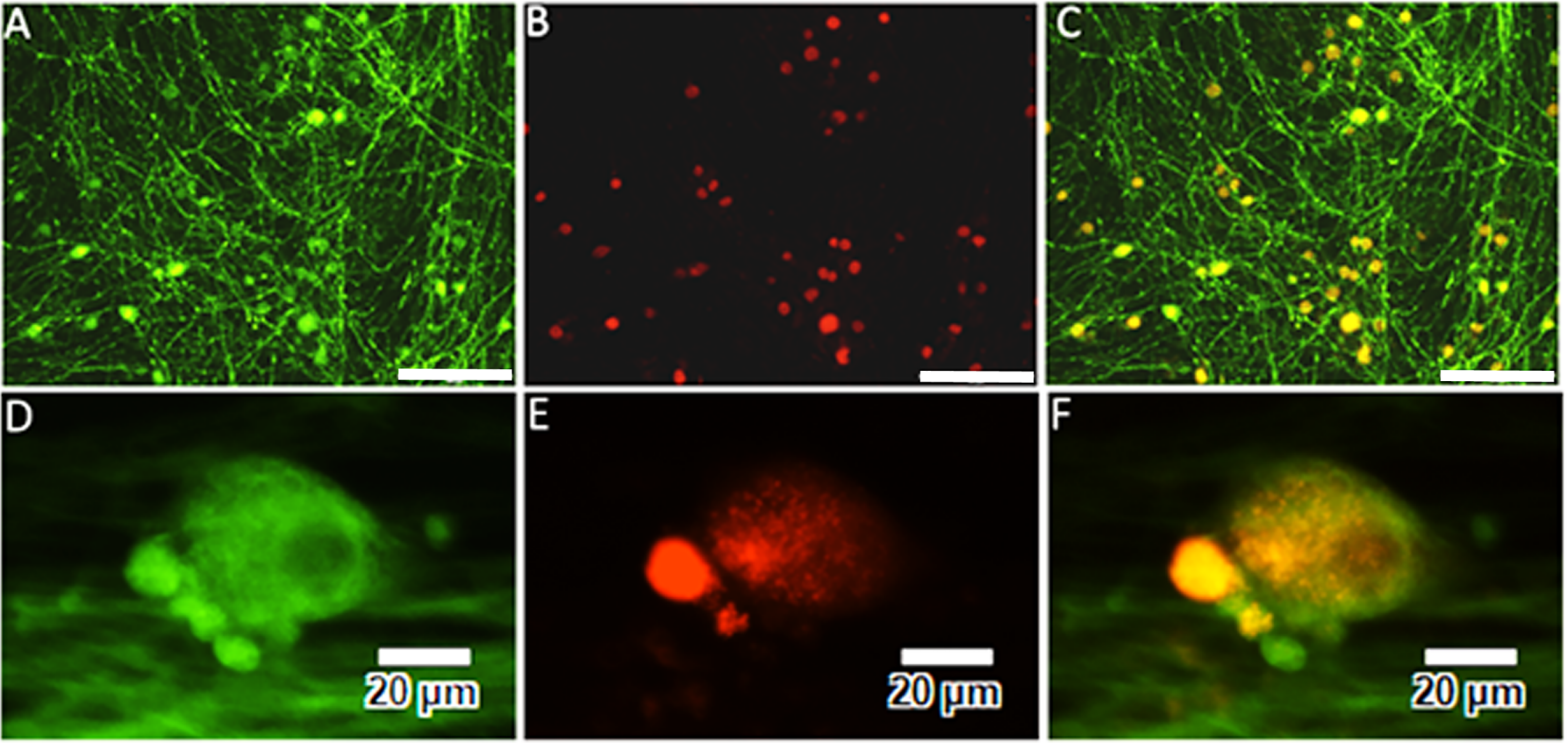
GLP-1R immunofluorescence in DRG neurons. Cultured adult rat DRG neurons (A, B, C) showing β tubulin immunofluorescence (green in A), GLP-1R expression (red in B), and the merged image (C). Bar = 200 μm. High power image of cultured human DRG neurons positive for β tubulin (green in D), intense GLP-1R expression (red) in a small neuron (E), and merged image (F). Bar = 20 μm.

GLP-1R expression was observed in majority of the cultured rat DRG neurons, including small and large diameter neurons (Fig 8A–8C). In cultured human neurons, smaller diameter neurons were more intensely positive for GLP-1R than large neurons (Fig 8D–8F).

**Fig 9:**
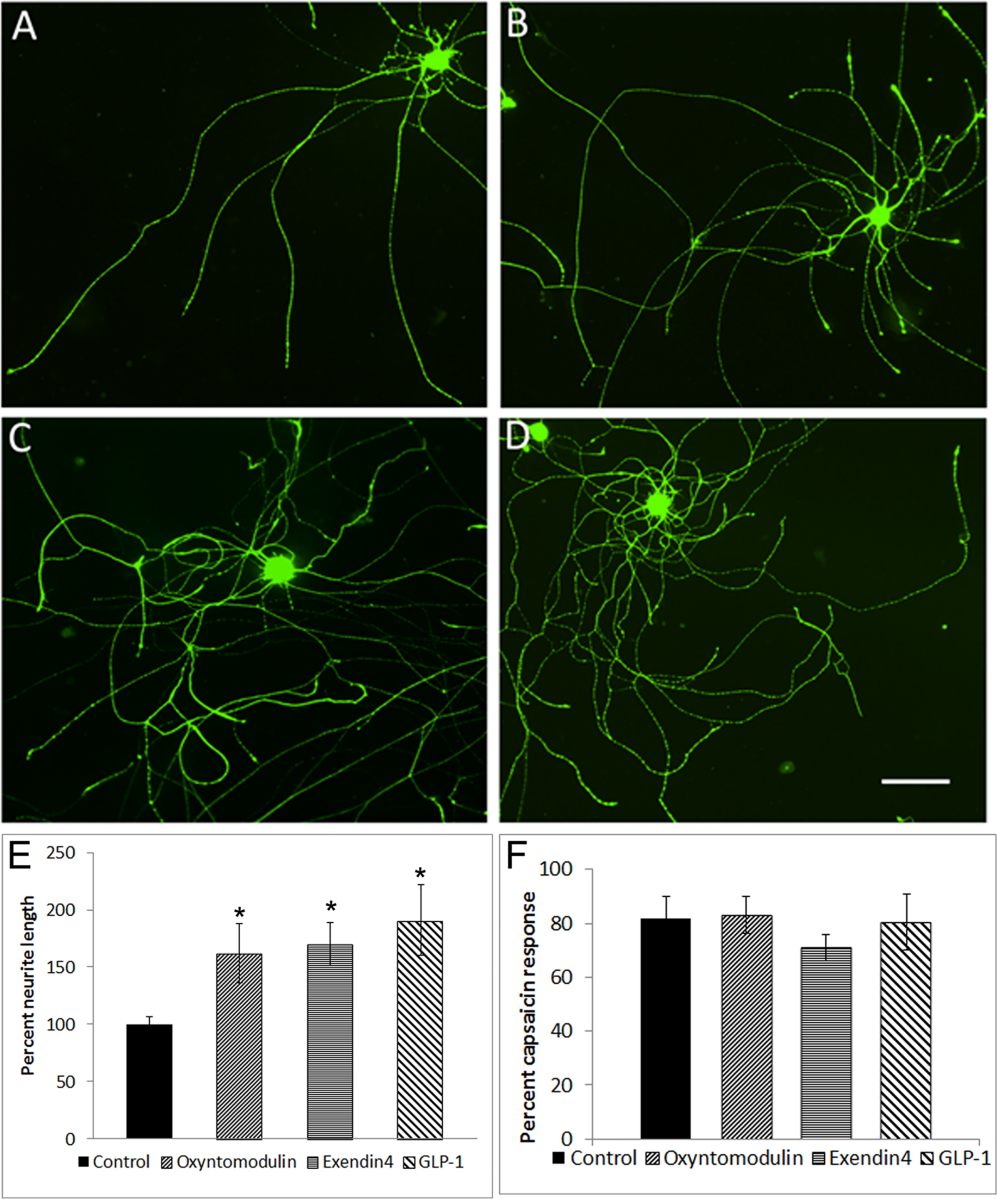
Effect on neurite outgrowth and capsaicin responses. Representative images showing β tubulin immunofluorescence in rat DRG neurons cultured with vehicle (A, control), with Oxm (B), exendin-4 (C), or GLP-1 (D). Bar = 100 microns. Graph showing significantly increased neurite lengths after 48 h treatment with oxyntomodulin (*p = 0.04), exendin-4 (*p = 0.01) and GLP-1 (*p = 0.04) using Student’s unpaired *t*-test (E). Graph showing lack of significant effect of acute treatment with incretin hormones on capsaicin sensitivity in adult rat DRG neurons (F).

Neurite length was increased in neurons treated with oxyntomodulin (*p = 0.04), exendin-4 (*p = 0.01) and GLP-1 (*p = 0.04), compared to controls (culture medium), (paired *t*-Test, data from n = 4 experiments, 30–50 neurons per treatment in each experiment (Fig 9A–9E). No toxic effects were observed in treated cultures at the concentrations used here.

**Fig 10:**
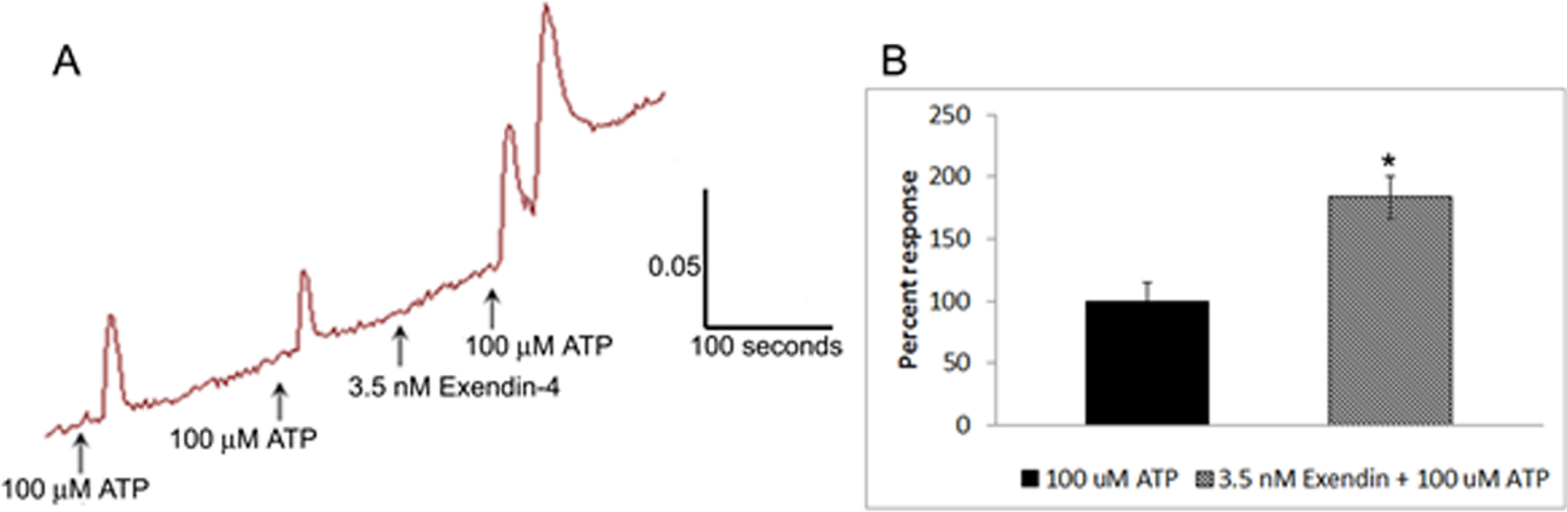
Effect of exendin-4 on ATP responses in human DRG neurons. Sample trace showing responses to ATP in the absence and presence of exendin-4 (A). Graph showing responses to ATP are significantly enhanced in the presence of exendin-4 (*p = 0.02, Student’s unpaired *t*-test) (B).

Application of the incretin hormones did not stimulate calcium influx. Capsaicin sensitivity was not significantly affected by acute treatment with 100 nM oxyntomodulin (15 neurons), exendin-4 (7 neurons), or GLP-1 (11 neurons), compared to controls (12 neurons), using calcium imaging (Fig 9F), but responses to 100 μM ATP were significantly enhanced in the presence of 3.5 nM exendin-4 (*p = 0.02, 4 neurons) (Fig 10).

## Discussion

This study shows that the GLP-1R-like immunoreactivity is expressed by the innervation of human colon and increased in biopsies from individuals with IBD (shown with two different antibodies), as are CGRP-positive sensory nerve fibres. Intense GLP-1R-like immunostaining was observed in small and medium-sized neuronal cell bodies and nerve fibres in human DRG. Specificity of GLP-1R antibodies was confirmed by positive staining in GLP-1R transfected HEK293 cells, but not in untransfected cells, or after omission of the primary antibody. Co-localization of GLP-1R expression with neuronal markers in the colon and DRG confirmed the neural expression of GLP-1R. This is the first report of GLP-1R expression in human DRG, and colon innervation in IBD.

GLP-1 stimulates insulin release from pancreatic beta cells, via a cAMP mediated pathway [35, 36] which also promotes neurite outgrowth in neurons [37], and may influence their sensitivity. The GLP-1R/CGRP positive nerve fibres observed in the normal and IBD colon sections include the sensory innervation responsible for conveying afferent signals via DRG neurons to the CNS in the gut-brain axis, besides the vagus nerve afferents. The combined results of increased nerve fibre density in IBD biopsies compared to controls, and increased neurite length after gut hormone treatment *in vitro*, indicates a potential role of nerve sprouting in the pathophysiology of IBD.

Primary afferents in the gut are polymodal, being activated by mechanical, thermal and chemical stimuli, via specific ion channels [38]. Small to medium-sized CGRP-containing colon primary afferents are found in rodent DRG with two peaks, at levels T8-L1 and L6-S1 [39]. A peripheral role for the GLP-1R is indicated by the reduction of food intake, gastric emptying, and insulin levels, in normal subjects following exogenous GLP-1 administration [17], effects that are lost in truncally vagotomised humans [40], and rats [41]. Modulating vagus nerve activity by electrical stimulation may thus be effective in weight loss and glycaemic control [42].

Nutrient chemosensing results in L cell-derived transient elevation of GLP-1, which may play a role in the maintenance of normal gut innervation [43, 44]. A recent study showed that higher disease activity in patients with IBD is associated with significantly increased plasma levels of GLP-1 and prolonged gastric emptying, confirming a correlation between the two; following effective therapy, gastric emptying was found to be accelerated and GLP-1 release decreased significantly [45]. In a separate study, the expression of dipeptidyl peptidase-4 (that inactivates GLP-1) was found to be significantly reduced in tissue and plasma from patients with active Crohn’s Disease, potentially elevating and enhancing the effects of GLP-1 [46]. As GLP-1 regulates appetite, elevated levels in IBD may contribute to the loss of appetite observed in these patients. In relation to our findings, chronically elevated levels of GLP-1 in IBD may provide a neurotrophic effect, resulting in increased nerve fibre density of GLP-1R expressing nerve fibres. The patients in our study all had gastrointestinal symptoms and gut dysmotility: further longitudinal studies are required to assess the relationship of the level of GLP-1R gut innervation to satiety and gut motility, and active disease.

Our findings extend the previous reports of GLP-1R co-localisation with nNos or ChAT in myenteric plexus neurons of humans [21,22] and mice [23], where it mediated inhibition of colonic contractility via a nitregic signalling pathway [21,22]. Relaxation caused by GLP-1 in gastric strips was abolished by L-NAME (nNOS inhibitor) and tetrodotoxin [35], indicating nitric oxide (NO) and neuronal voltage-dependent Na^+^ channel involvement respectively, in maintenance of gut motility.

GLP-1R immunofluorescent neurons were observed in cultures of human and rat DRG, and 48 hour treatment of cultured rat neurons with oxyntomodulin, exendin-4 and glucagon resulted in significantly increased neurite lengths, compared with controls. These observations are in agreement with previous reports of neural GLP-1R expression [26–28, 31], and neurite promoting effects of GLP-1R agonists [29]. The absence of calcium influx in DRG neurons following the acute application of these gut hormones, observed in our study, was also reported in mouse nodose ganglion neurons which express GLP-1R, whereas GLP-1 was reported to enhance ATP responses and increase firing frequency of cultured mouse enteric neurons [31]. In agreement with this report, our findings showed that ATP responses were significantly enhanced in human DRG neurons in the presence of the synthetic GLP-1 analogue, Exendin-4. We have previously shown expression of P2X3 receptor (which is activated by ATP) in enteric neurons of the submucous plexus in the normal human gut, with an increase in patients with active CD [47].

Our study showed that the nociceptive capsaicin / heat pain receptor TRPV1 was not affected by acute application of these gut hormones, indicating that the GLP-1R may not participate in noxious signalling involving TRPV1. A recent clinical trial of a GLP1 analog ROSE-010, reported reduction of pain during acute exacerbations of irritable bowel syndrome, and increased colonic transit providing relief of constipation [48]. These observations, combined with our findings of enhanced ATP signalling in the presence of Exendin-4 in the human DRG neurons, and by others in rodent neurons [31], suggest that GLP-1R may participate in modulating gut motility, rather than pain signalling. However, comparisons between short and long term exposure of GLP-1R agonists in the presence of the GLP-1R antagonist Exendin-9, on neurite outgrowth, neuronal sensitivity and TRPV1 expression in thoracic and lumbosacral DRG are required, and will be the focus of future studies. Nerve growth factor (NGF) is currently regarded as the major regulator of TRPV1 expression and hypersensitivity in nociceptors and inflamed bowel [1, 4, 34]. The neurite promoting effects combined with the lack of sensitization of capsaicin responses suggests GLP-1R agonists may be beneficial in other conditions such as diabetic neuropathy, with the potential to provide neurite regeneration without the burden of nociceptor sensitization or enhancement of pain.

In conclusion, GLP-1R is expressed in nerves innervating the intestine, and is significantly increased in bowel biopsies from IBD patients. The incretin hormones promote neurite outgrowth in cultured DRG neurons, and may underlie the observation of increased nerve fibres in IBD. While ATP signalling was enhanced, capsaicin sensitivity was not affected by acute application of exendin-4 or oxyntomodulin in sensory neurons. These studies show increased GLP-1R innervation may be involved in IBD bowel pathophysiology and afferent signalling, and potentially provide a peripheral therapeutic target for satiety and weight loss.

## Supporting information

S1 Fig Specificity of GLP1-R immunostaining in GLP-1R transfected HEK cell lines. Staining in HEK negative cell line (A, C) or in GLP-1R transfected HEK cell line (B, D) using GLP-1R Abcam antibody at a dilution of 1:1000 or at 1:2000, magnification x40.
(TIF)

S2 Fig Specificity of GLP1-R immunostaining in GLP-1R transfected HEK cell lines. Staining in HEK negative cell line (A, C, E) or in GLP-1R transfected HEK cell line (B, D, F) using GLP-1R Acris antibody at a dilution of 1:100, 1:200 and at 1:500, magnification x40.
(TIF)

## Abbreviations

GLP-1R: Glucagon-like peptide 1 receptor
IBD: Inflammatory bowel disease
DRG: dorsal root ganglion
CD: Crohn’s disease
UC: ulcerative colitis
PGP9.5: protein gene product 9.5
CGRP: Calcitonin gene-related peptide
ATP: adenosine triphosphate
DPP: dipeptidyl peptidase
