# Supplementary figures and images for: Glucagon-like peptide 1 receptor (GLP-1R) expression by nerve fibres in inflammatory bowel disease and functional effects in cultured neurons
Source: PLoS One. 2018 May 29;13(5):e0198024. doi: 10.1371/journal.pone.0198024 (PMC5973579; doi:10.1371/journal.pone.0198024)

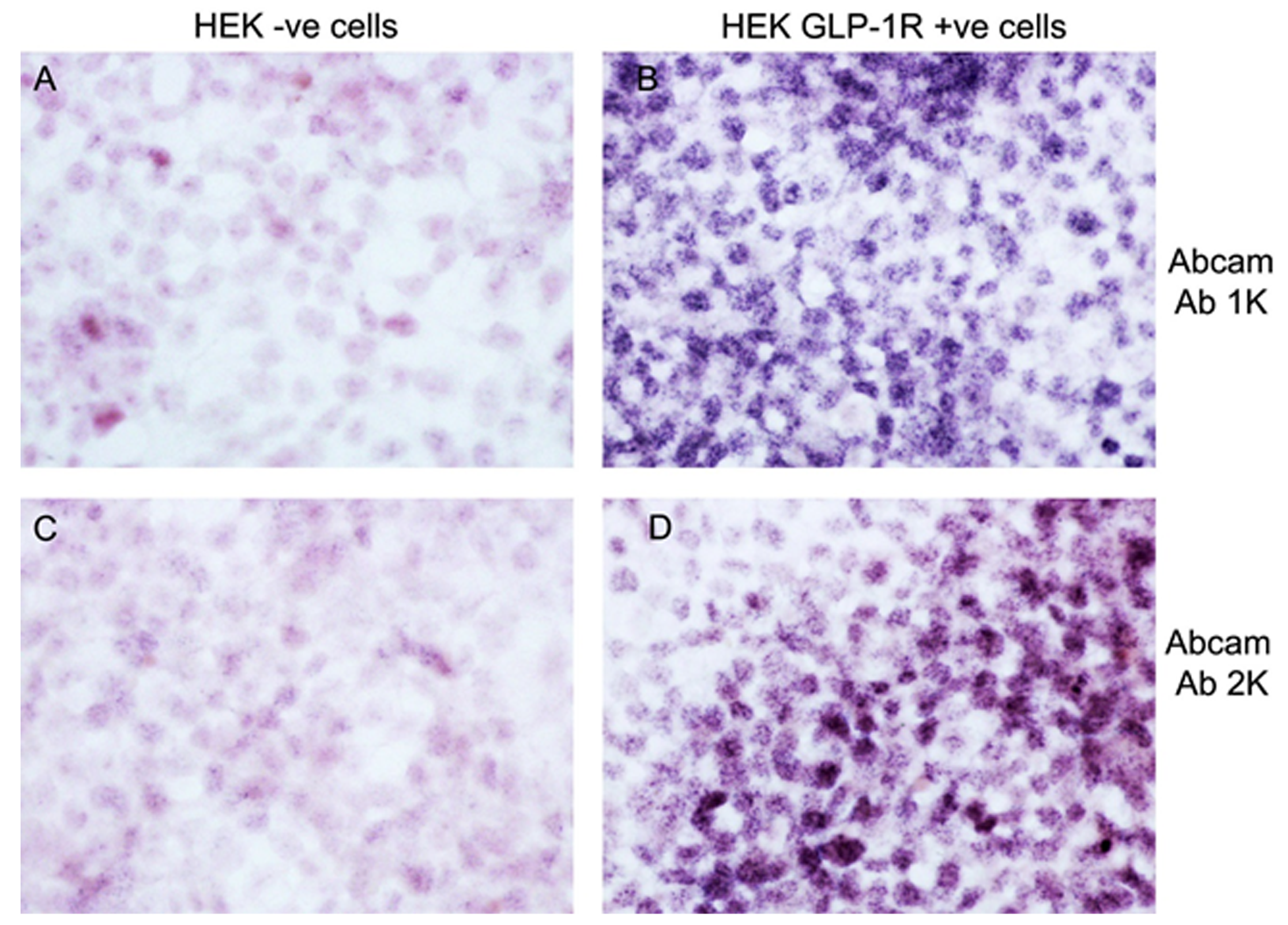

Supplement: S1 Fig — Staining in HEK negative cell line (A, C) or in GLP-1R transfected HEK cell line (B, D) using GLP-1R Abcam antibody at a dilution of 1:1000 or at 1:2000, magnification x40. (TIF) [file pone.0198024.s001.tif]

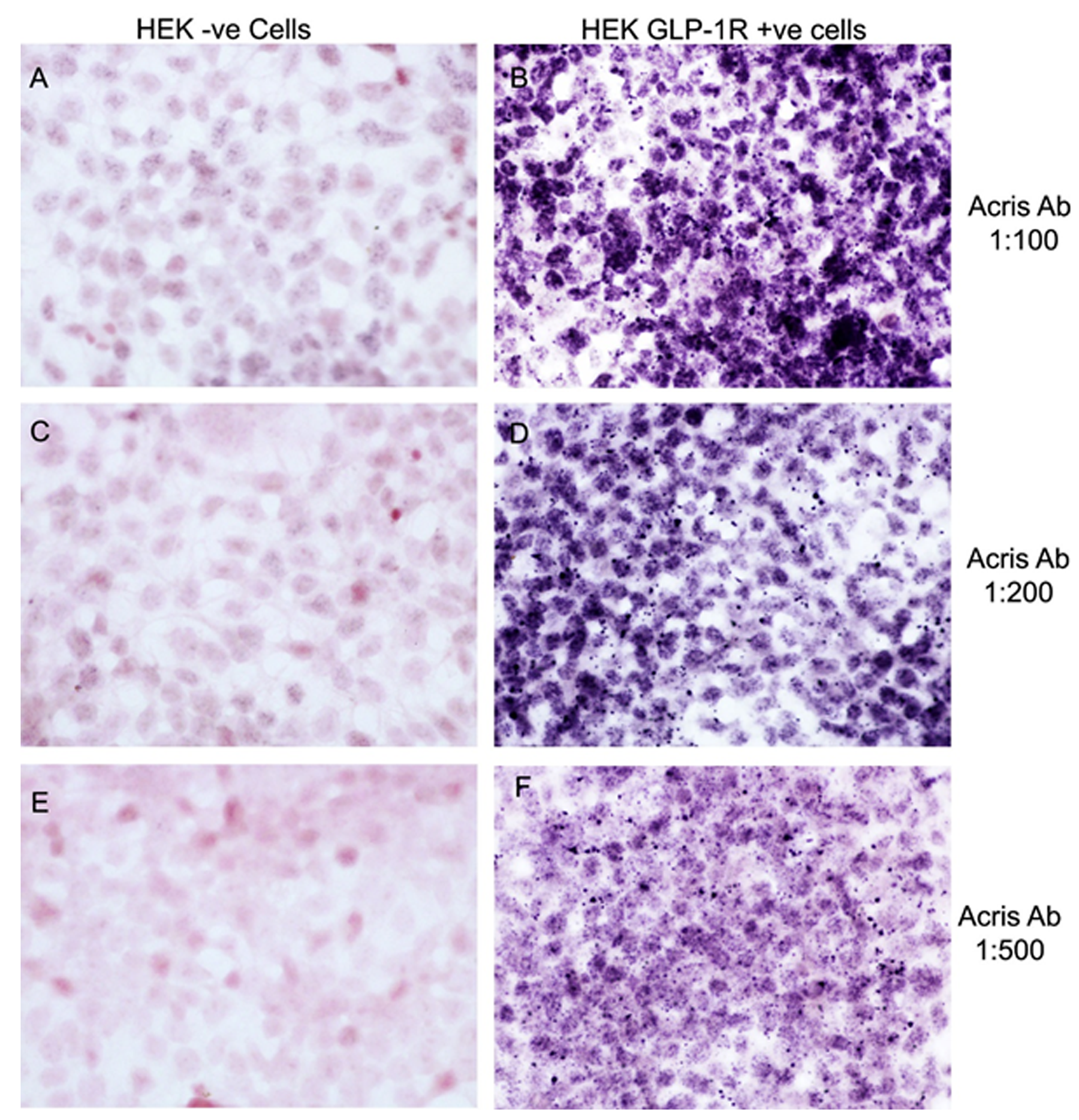

Supplement: S2 Fig — Staining in HEK negative cell line (A, C, E) or in GLP-1R transfected HEK cell line (B, D, F) using GLP-1R Acris antibody at a dilution of 1:100, 1:200 and at 1:500, magnification x40. (TIF) [file pone.0198024.s002.tif]
